# Supplementary material for: MetaQTL: a package of new computational methods for the meta-analysis of QTL mapping experiments
Source: BMC Bioinformatics. 2007 Feb 8;8:49. doi: 10.1186/1471-2105-8-49 (PMC1808479; doi:10.1186/1471-2105-8-49)
Supplement: Additional File 7 — MetaQTL Package : jar file and tutorial. This Zip archive contains both the MetaQTL JAR file and the files of the tutorial. [file 1471-2105-8-49-S7.zip › org.inra.metaqtl/doc/org/metaqtl/EMResult.html]

EMResult


|  |  |  |
| --- | --- | --- |
| **Overview** \| **Package** \| **Class** \| **Use** \| **Tree** \| **Deprecated** \| **Index** \| **Help** | |  |
| **PREV CLASS**   **NEXT CLASS** | **FRAMES**    **NO FRAMES**     **All Classes** |  |
| SUMMARY: NESTED \| FIELD \| CONSTR \| METHOD | DETAIL: FIELD \| CONSTR \| METHOD |  |


---


## org.metaqtl Class EMResult

```
java.lang.Object
  org.metaqtl.EMResult
```

---

``` public class EMResult extends java.lang.Object ```

---

| **Field Summary** | |
| --- | --- |
| `double[]` | `ccov`             The complete information (just for means). |
| `double` | `clog`             The complete loglikelihood. |
| `EMCriteria` | `criteria`             The model choice criteria. |
| `double[][]` | `dm`             The matrix of convergence rate for the EM-Algorithm (just for means). |
| `double` | `edist`             The euclidean distance between the current and the previous vector of parameters (mu + pi) |
| `int` | `k`             The number of clusters. |
| `double[]` | `mu`             The means of the clusters. |
| `int` | `n`             The number of data points. |
| `double[][]` | `ocov`             The observed information (just for means). |
| `double` | `olog`             The observed loglikelihood. |
| `double[]` | `pi`             The mixing proportions. |
| `double` | `rate`             The quadratric rate of convergence of the algorithm. |
| `double[][]` | `z`             The cluster membership probabilties. |


**Constructor Summary**

`EMResult()`

`EMResult(int n,
int k)`


| **Method Summary** | |
| --- | --- |
| `void` | `computeCriteria()`             This methos computes the following criteria : AIC AIC3 ICOMP MIR BIC AWE |
| `static void` | `copy(EMResult dest, EMResult src)` |
| `double` | `getCriterion(int critIdx)` |
| `double[]` | `getEuclidean()` |
| `int` | `getK()` |
| `double[]` | `getMahalanobis(double[] sd)` |
| `double` | `getMu(int i)` |
| `double` | `getPi(int i)` |
| `double` | `getSD(int i)` |
| `double[]` | `getXBestPred()` |
| `double[]` | `getXPred()` |
| `int` | `getXPredIdx(int i)` |
| `double[]` | `getZ(int i)` |
| `void` | `sortCluster()`             Sort the components in increasing order. |

**Methods inherited from class java.lang.Object**

`clone, equals, finalize, getClass, hashCode, notify, notifyAll, toString, wait, wait, wait`

**Field Detail**

### k

```
public int k
```

:   The number of clusters.

---


### n

```
public int n
```

:   The number of data points.

---


### rate

```
public double rate
```

:   The quadratric rate of convergence
    of the algorithm.

---


### edist

```
public double edist
```

:   The euclidean distance between the
    current and the previous vector of
    parameters (mu + pi)

---


### mu

```
public double[] mu
```

:   The means of the clusters.

---


### pi

```
public double[] pi
```

:   The mixing proportions.

---


### z

```
public double[][] z
```

:   The cluster membership probabilties.

---


### olog

```
public double olog
```

:   The observed loglikelihood.

---


### clog

```
public double clog
```

:   The complete loglikelihood.

---


### dm

```
public double[][] dm
```

:   The matrix of convergence rate
    for the EM-Algorithm (just for means).

---


### ocov

```
public double[][] ocov
```

:   The observed information (just for means).

---


### ccov

```
public double[] ccov
```

:   The complete information (just for means).

---


### criteria

```
public EMCriteria criteria
```

:   The model choice criteria.


**Constructor Detail**

### EMResult

```
public EMResult()
```

---


### EMResult

```
public EMResult(int n,
                int k)
```

**Parameters:**: `n` - the number of data points.: `k` - the number of components.


**Method Detail**

### sortCluster

```
public void sortCluster()
```

:   Sort the components in increasing order.

---


### copy

```
public static void copy(EMResult dest,
                        EMResult src)
```

:   **Parameters:**: `theta` -: `spoint` -

---


### computeCriteria

```
public void computeCriteria()
```

:   This methos computes the following criteria :

    - AIC
    - AIC3
    - ICOMP
    - MIR- - BIC
        - AWE

---


### getCriterion

```
public double getCriterion(int critIdx)
```

:   **Parameters:**: `critIdx` -

---


### getMahalanobis

```
public double[] getMahalanobis(double[] sd)
```

:   **Parameters:**: `sd` -

---


### getXPred

```
public double[] getXPred()
```

:   **Returns:**

---


### getSD

```
public double getSD(int i)
```

:   **Parameters:**: `i` - **Returns:**

---


### getMu

```
public double getMu(int i)
```

:   **Parameters:**: `i` - **Returns:**

---


### getK

```
public int getK()
```

:   **Returns:**

---


### getZ

```
public double[] getZ(int i)
```

---


### getEuclidean

```
public double[] getEuclidean()
```

:   **Parameters:**: `sd` - **Returns:**

---


### getPi

```
public double getPi(int i)
```

:   **Parameters:**: `i` - **Returns:**

---


### getXPredIdx

```
public int getXPredIdx(int i)
```

:   **Parameters:**: `i` - **Returns:**

---


### getXBestPred

```
public double[] getXBestPred()
```

:   **Returns:**


---


|  |  |  |
| --- | --- | --- |
| **Overview** \| **Package** \| **Class** \| **Use** \| **Tree** \| **Deprecated** \| **Index** \| **Help** | |  |
| **PREV CLASS**   **NEXT CLASS** | **FRAMES**    **NO FRAMES**     **All Classes** |  |
| SUMMARY: NESTED \| FIELD \| CONSTR \| METHOD | DETAIL: FIELD \| CONSTR \| METHOD |  |


---
